# Maternal methylmercury exposure during early-life periods adversely affects mature enamel structure of offspring rats at human exposure levels: a concern for oral health

**DOI:** 10.3389/fpubh.2023.1183308

**Published:** 2023-06-30

**Authors:** Victória Santos Chemelo, Leonardo Oliveira Bittencourt, Priscila Cunha Nascimento, Mayra Frasson Paiva, Alberto Carlos Botazzo Delbem, Juliano Pelim Pessan, Alexandre Ribeiro do Espírito Santo, Alan Rodrigo Leal Albuquerque, Rômulo Simões Angélica, Maria Elena Crespo-Lopez, Sofia Pessanha, Michael Aschner, Rafael Rodrigues Lima

**Affiliations:** ^1^Laboratory of Functional and Structural Biology, Institute of Biological Sciences, Federal University of Pará (UFPA), Belém, Pará, Brazil; ^2^Department of Preventive and Restorative Dentistry, School of Dentistry, São Paulo State University (UNESP), Araçatuba, Brazil; ^3^Laboratory of Histotechnology and Tissue Biology, Department of Biomorphology, Institute of Health Sciences, Federal University of Bahia (UFBA), Salvador, Bahia, Brazil; ^4^Laboratory of X-Ray Diffraction, Institute of Geosciences, Federal University of Pará, Belém, Pará, Brazil; ^5^Laboratory of Molecular Pharmacology, Institute of Biological Sciences, Federal University of Pará (UFPA), Belém, Pará, Brazil; ^6^Laboratory of Instrumentation, Biomedical Engineering and Radiation Physics, NOVA School of Science and Technology, Caparica, Portugal; ^7^Department of Molecular Pharmacology, Albert Einstein College of Medicine, Bronx, NY, United States

**Keywords:** enamel, methylmercury, offspring, development, toxicity

## Abstract

Although there are many studies on the health effects of methylmercury (MeHg) toxicity during *in utero* and early development, little is known about its effects on mineralized tissues present in the oral cavity, such as enamel structure. Therefore, this study evaluated the effects of MeHg exposure on the physico-chemical, ultrastructural and functional properties of mature tooth enamel. Specifically, we studied offspring of mothers exposed to MeHg during the prenatal and postnatal periods which are the developmental stages associated with tooth enamel formation. Female rats were exposed to MeHg at a dose of 40 μg/kg/day for 42 days of pregnancy and lactation. The enamel of offspring was analyzed by (1) Fourier Transform Infrared Spectroscopy and Raman to assess physicochemical composition, (2) Scanning Electron Microscopy for ultrastructural evaluation, (3) Transmitted Polarizing Light Microscopy for analysis of the enamel extracellular matrix, and (4) resistance and hardness were evaluated by microhardness. The results showed that MeHg exposure during this sensitive enamel formation period induced changes in inorganic and organic content and enamel prisms ultrastructure alterations and disturbed the organic extracellular matrix due to a decreased enamel strength. These novel findings establish for the first time that maternal exposure to MeHg pre and postnatal promoted relevant changes in mature enamel of their offspring rats.

## 1. Introduction

Mercury is a ubiquitous toxic metal in the environment, which continues to pose public health concerns worldwide (1). Mercury is currently one of the top 10 identified public health hazardous chemicals (2, 3). Mercury species are mainly classified into three groups: metallic (Hg^0^), inorganic (Hg^2+^), and organic mercury [CH_3_Hg^+^, (CH_3_)_2_Hg, etc.] (2, 4), with the metallo-organic methylmercury (MeHg) being particularly toxic to humans because of its toxicokinetic properties. MeHg is primarily formed in the environment upon methylation of Hg^+2^ by methanogenic and sulfate-reducing bacteria (3, 5). In addition, human artisanal and small-scale gold mining (ASGM) leads to the release of mercury into the environment, especially MeHg (3, 6, 7).

Human exposure may occur in occupational settings, such as the use of the metal in odontology practice or ASGM, when predominantly Hg^0^ is inhaled, or by environmental routes when MeHg is ingested through contaminated food (6, 8). This metal has soluble complexes mainly linked to the sulfur atom of the thiol binders (9). MeHg is absorbed in the gastrointestinal tract and distributed into the blood and is demethylated for a long period to mercuric Hg in tissues, including in the fetal liver (10). In the latter case, the propensity of MeHg to bioaccumulate and biomagnify through the food chain, leads to chronic exposures in populations and pose risks to human health (3, 6). After ingestion, MeHg leads to physiological and biochemical alterations in various human organs (11). Evidence of MeHg toxicity to neurological, immunological, cardiovascular, and reproductive pathways in humans is widespread in the literature. Studies from our research group have demonstrated effects on the motor cortex, cerebellum, hippocampus, alveolar bone and salivary glands (12–17).

MeHg toxicity is also of major concern upon fetal and neonatal exposures, posing risk to human development due to the ability of MeHg to readily cross the placenta and the blood–brain barriers, and its transport into breast milk during the lactation period (18, 19). Recently, structures associated with the oral cavity have been affected by organic mercury exposure, including the salivary glands, alveolar bone and periodontal ligament and pulp stem cells (15, 17, 20–22). However, as far as mineralized tissues, such as bone and teeth, studies on the effects of MeHg are scant, both in adults and neonates.

Tooth enamel is an embryonic epithelial-derived tissue, acellular and irreparable surface layer, which has no physiological means of repair (23), with remarkable characteristics of supporting masticatory forces, at the same time capable of protecting the tooth structure from external variations and participate in the dynamic demineralization-remineralization process (24–26), emphasizing the great importance of this tissue for maintaining oral cavity homeostasis. *In utero* environmental exposures of the developing offspring can cause oral abnormalities, such as tooth malformations, which makes the tooth more susceptible to dental caries (27, 28). Of all the childhood dental diseases, dental caries remains one of the most prevalent (29). More than 530 million children worldwide have untreated caries in the primary dentition, with the prevalence of the disease increasing with age (30). In this way, structural damage to enamel, even during its formation, is irreparable and generally associated with increased susceptibility to secondary injury (31). In rodents, enamel formation commences in the embryonic period, enabling a suitable translational correlation with deciduous teeth in humans (32–34).

To date, there no evidence has been advanced in associating MeHg exposure with aberrant enamel homeostasis. Accordingly, this study aimed to evaluate the effects of maternal exposure to MeHg on the physicochemical, structural, and functional properties of mature dental enamel in offspring rats.

## 2. Materials and methods

### 2.1. Animals and experimental groups

Eight pregnant rats, *Rattus norvegicus*, 90 days old, weighing 250–300 g, were randomly divided into two experimental groups (*n* = 4 per group). Identification of the genital plug coincided with embryonic day 1. During the gestational and lactational periods, rats were randomly kept in polypropylene cages (1 per cage), with *ad libitum* access to food and distilled water, in an acclimatized room (25 ± 2°C) on a 12-h light/dark cycle. An average of 2 males were born for every female rat throughout pregnancy, and 1 female gave birth to an average of 5 males every litter. No deaths and exclusions were identified throughout the gestational period. The animals were provided and delivered by the Central Animal Facility of the Federal University of Para, under a protocol of the Ethics Committee on Animal Use No. 8613011217 (CEUA/UFPA last approval date in 11/22/2019) and followed the ARRIVE 2.0 guidelines (35). All the procedures of animal care followed the Guide for the Care and Use of Laboratory Animals (36).

### 2.2. Methylmercury administration

The procedure for MeHg exposure was performed by dissolving MeHg chloride (Sigma-Aldrich, United States) in ethanol (vehicle) and incorporating it into cookies (Teddy Grahams, Nabisco, Canada) in order to achieve a dosage of 40 μg/kg/day (37), for 42 days, according to the protocol outlined in previous studies (38, 39).

For this purpose, each dam was weighed weekly for dose calculation, and the appropriate amount of solution was placed on the cookie and subsequently dried at room temperature. The control group received cookies with vehicle only, in the same proportional volume and for the same period. The cookies were offered once a day, individually to the rats. The postnatal period is characterized by lactation, which in rats lasts an average of 21 days. During both periods (pregnancy and lactation), only the mother received the treatment. Our experimental design consisted of two moments: the first, when the dams were directly exposed to the cookies (with or without MeHg) during the 21 days of pregnancy, and the lactation until 21-day of life; and the second, which did not involve MeHg exposure to the dam or to the offspring. The animals were kept separated until the 41 days of life, which is the period between adolescence and early adulthood (40–42). This period corresponds to the animal’s incisors developmental stages (32, 33).

### 2.3. Sample collection

After the gestational and lactational MeHg-exposure periods, the offspring were divided by sex and kept in collective cages following the guidelines of experimental animal care, as previously described (36). At 41 days of life, the offspring were anesthetized via intraperitoneal with ketamine hydrochloride (180 mg/kg) and xylazine hydrochloride (30 mg/kg), euthanized, and the maxillary incisors were collected.

A set of animals (*n* = 10 per group) were perfused with heparinized saline solution (0.9%) and fixed with 4% formaldehyde. Hemimaxillae covering posterior portion of one upper incisor from 8 rats from each group were decalcified by immersion in aqueous solution of 5% nitric acid and 4% formaldehyde (under constant shaking for 24 h). After dehydration and clarification, demineralized samples were paraffin-embedded, and 5 μm-thick longitudinal serial sections were obtained using a Leica RM2125 microtome (Leica Microsystems, Wetzlar, Germany). Twenty longitudinal sections of hemimaxillae exhibiting buccal secretory stage enamel organic extracellular matrix (EOECM) of upper incisor were carefully chosen, using as parameter the parallelism of the cut along the long axis of the sample. Therefore, sections showing lateral secretory stage enamel were rejected and the secretory stage enamel of all the chosen sections from each group presented similar height and equivalent matrix proteins arrangement, and thus the groups could be compared as for their birefringence. After removal of the paraffin with xylene and hydration, the sections were analyzed by transmitted polarizing light microscopy (TPLM).

**Figure 1:**
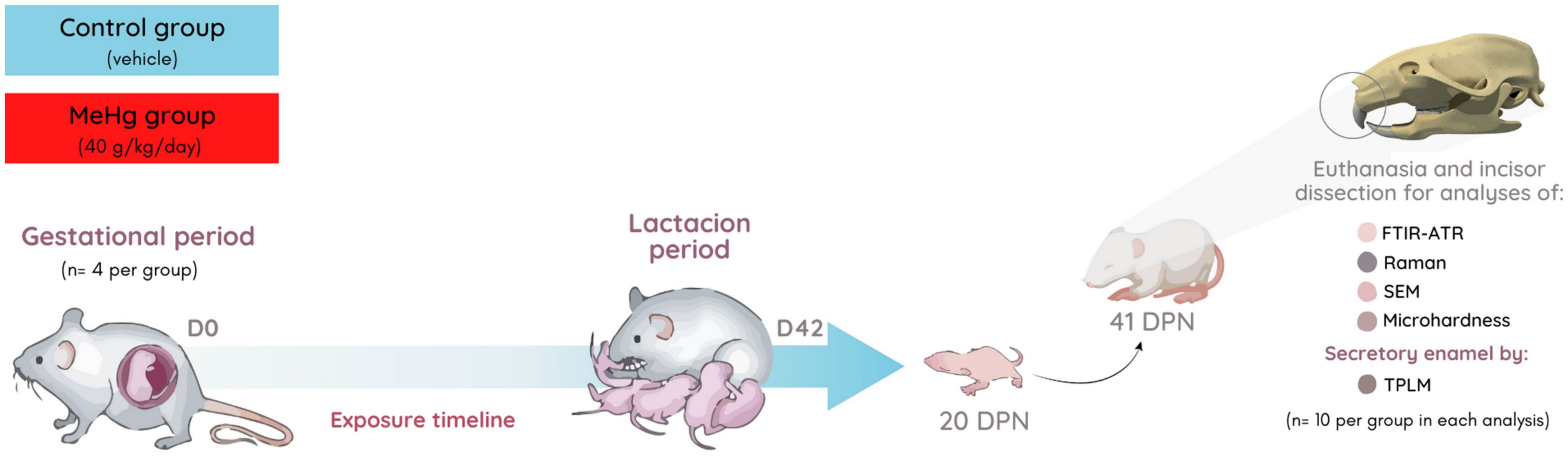
Sample description and experimental stages. Pregnant rats’ exposure to MeHg (40 μg/kg/day) during pregnancy and lactation periods (42 exposure days). The incisors of offspring were collected for mature enamel analysis by physical–chemical (FTIR-ATR), phosphate content (Raman), ultraestructural (SEM), microhardness analyzes, and secretory stage enamel organic extracellular matrix (TPLM). In each evaluation, right/left hemimaxillary incisors were used (*n* = 10 per group). DPN, days postnatal.

Left side incisors (*n* = 10 per group) were used for SEM analysis, while right side incisors were used for hardness analysis. Once perfused, the 20 other teeth incisors (*n* = 10 per group) were collected for TPLM analyzes (right side). The remaining incisors were used for FTIR-ATR and Raman analyzes (left side). The experimental design is summarized in Figure 1.

### 2.4. Physico-chemical properties of enamel analysis

To determine possible physico-chemical changes in enamel from the vibrational modes of phosphate, carbonate and amide, present in the mineral and organic component of the tissue, the enamel surface of the incisal edge was analyzed by infrared spectrometry, obtained by attenuated total reflectance (ATR), using a Thermo spectrometer, model Nicolet iS50 FT-IR, in the 4,000–400 cm^−1^ spectral region, at 100 scans and 4 cm^−1^ resolution. As a pre-treatment, the samples were dried at room temperature for 4 h. Data acquisition was performed using OMNIC software from the spectra record and the results were determined by integrating the average area found under the bands of the samples of the respective groups.

### 2.5. Phosphate mineral content analysis

To determine the possible crystalline structure changes from symmetric stretching band of phosphate, Raman spectra were obtained with a Horiba XploRA Confocal Microscope using the near infrared laser (785 nm) with a 1,200 line/mm grating. Thus, the spectral range investigated was from 300 cm^−1^ to 1800 cm^−1^ with a spectral resolution of 4 cm^−1^. Using an entrance slit of 100 μm and a confocal hole of 300 μm, the scattered light collected by the objective was dispersed onto the air-cooled CCD array of an Andor iDus detector. A 100× objective (N.A. = 0.9) was used to focus on the enamel surface, as well as a 50% neutral density filter rendering an incident power on the sample of 5.0 ± 0.4 mW (lasercheck®, Edmund optics).

Spectra were obtained by three accumulations of 20 s each and an average of five measurements were performed on each sample. To determine the depolarization ratio (ρ) of the most intense band in the Raman spectrum, assigned to the symmetric stretching band of phosphate ions (ν_1_ ~ 959 cm^−1^), in each spot, spectra were recorded in two orthogonal polarizations of scattered light (perpendicular and parallel to the polarization of the incident laser). The ρ959 was then determined according to (43):


$$\rho_{959}=\frac{I_{959}⊥}{I_{959}II}$$

where $I_{959}\;II$ is the intensity of the Raman band at ~959 cm^−1^ using parallel polarization and $I_{959}⊥$ is the intensity of the Raman band at ~959 cm^−1^ using perpendicular polarization between the incident laser and the scattered radiation.

Moreover, unpolarized spectra were recorded and the ratio of the symmetric stretching and bending modes of phosphate (959/430–449 cm^−1^) and phosphate to carbonate (959/1070 cm^−1^) ratios were calculated.

Spectral deconvolution was performed using the software LabSpec (v5.58.25, Horiba, France), making use of a linear baseline correction to remove the background due to fluorescence. The intensities were determined by integrating the area under the bands.

### 2.6. Ultrastructural analysis

For ultrastructural analysis using scanning electron microscopy (SEM), incisors from the control and exposed groups were used. The incisal ridge was sectioned transversely with a carborundum disk, assembling in a straight handpiece and under irrigation, forming blocks with dimensions of 5 × 5 mm. The samples were sanded under irrigation with sandpaper sheets (3 M, Brazil) with granulation # 2000 and # 2500 and polished with felt disk, mounted on a straight handpiece, and polishing paste (3 M, Brazil). Cross sections of mature enamel were obtained from the tooth portion below the alveolar bone crest, not exposed to masticatory friction. Subsequently, all blocks were washed in distilled water, in an ultrasonic bath for 1 min. After drying, they were immersed in sodium hypochlorite at 1% for 5 min and washed again in an ultrasonic bath with distilled water for 30 s. Next, they were immersed in a 17% EDTA solution for 10 s, to remove micro debris originating from the cleavage and polishing process, and again washed in an ultrasonic bath for 1 min.

Each specimen was immersed for 5 min in an ascending series of alcoholic solutions (70, 90%, and absolute ethanol alcohol) and subsequently dried at room temperature (44). The samples were assembled, metallized, and observed using a scanning electron microscope (LEO-1430; Carl Zeiss, Germany). The micrographs were obtained in several regions of the incisal enamel: area at magnifications of 1,500× and 4,000 × .

### 2.7. Secretory enamel organic extracellular matrix analysis

Unstained longitudinal sections of one hemimaxilla from 10 rats from each group were analyzed by TPLM to determinate the optical retardation (nm) of birefringent brightness in the secretory stage EOECM of the maxillary incisor. The incisor tooth of each hemimaxilla was sectioned transversally at 2 mm above the alveolar bone crest, using a hard tissue microtome (South Bay Technology Inc., Model 650, United States). Twenty sections from each incisor were immersed in an 80% aqueous glycerin imbibing medium for 30 min. Five measurements of optical retardation were performed by a viewer blind to the investigated groups. A mean value for optical retardations was calculated for each animal (8 mean values were obtained from each group). Leica DM LP microscope (Leica Microsystems), polarizing filters, Brace-Köhler compensator (Wild Leitz, Wetzlar, Germany), and polychromatic light were used. Twenty-five percent of the sections studied with TPLM were stained with hematoxylin and eosin (HE) and analyzed with bright field light microscopy (BFLM) for confirming satisfactory structural preservation.

### 2.8. Microhardness analysis

**Figure 2:**
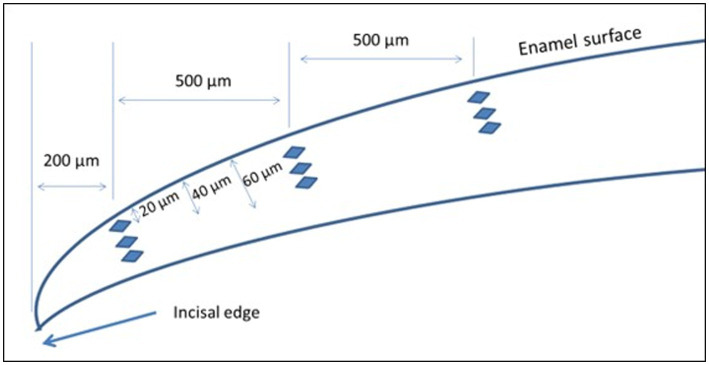
Schematic representation of cross-sectional hardness measurements.

The incisors were embedded in acrylic resin and the specimens were ground, polished and submitted to microhardness analysis in longitudinal sections, employing the microhardness meter Shimadzu HMV-2.000TM, with a Knoop indenter under a static load of 15 g for 10 s (45). In brief, three sequences of indents were performed at 20, 40 and 60 mm from the external enamel surface. The first sequence was made at 200 μm of the incisal edge, and the others at 500 μm from each other (Figure 2). The data were used for the calculation of mean cross-sectional hardness (KHN) and integrated area of hardness in depth (ΔKHN).

### 2.9. Statistical analyzes

The data distribution was tested by the Shapiro–Wilk method for verification of normality, and then analyzed by Student’s *t*-test (parametric data), except for the analysis of enamel organic extracellular matrix, where the Mann–Whitney test (non-parametric data) was used, with *p* values set at <0.05. The results were expressed as the mean ± standard error of mean or median and interquartile deviation, according to the normality of data.

## 3. Results

### 3.1. Maternal exposure to MeHg changes the inorganic and organic content iof the enamel structure of rats

**Figure 3:**
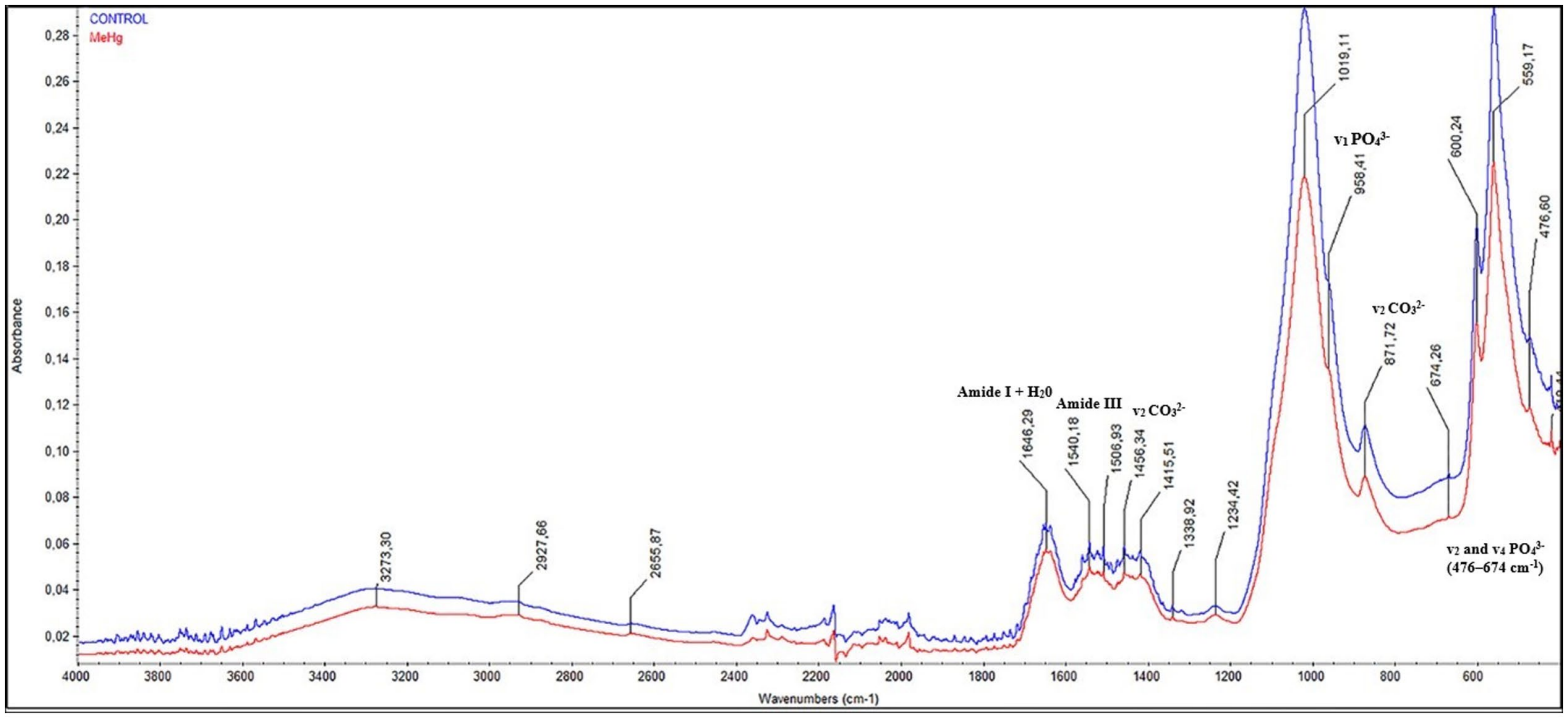
Effects of maternal MeHg exposure (40 μg/kg/day) and transfer to the offspring during the pre- and postnatal periods on FTIR infrared spectroscopic profile analysis in the enamel of the incisors of offspring rats (41 days old). The qualitative results were expressed by absorbance as a function of wavelength (cm^−1^), assigned to vibrations mode of PO4^3−^, CO3^2−^, amide I and III, in the comparison between the FTIR spectra of the control group (blue line) with that of the exposed group (red line).

In infrared spectrometry, obtained by ATR, animals exposed during maternal MeHg exposure (see Figure 3, red line) showed a decrease in the absorbance of bands assigned to phosphate (PO_4_^3−^), carbonate (CO_3_^2−^), and two types of amides. In the graphic, there were changes in the v_2_ and v_4_ vibrations of PO_4_^3−^ in the range of 476–674 cm^−1^ and v_1_ vibration in 958 cm^−1^ band. In the ion CO_3_^2−^, differences were evident in vibrational modes, v_2_ and v_3_, in the bands of 871 cm^−1^ and 1,415–1,456 cm^−1^, respectively. Finally, there were changes in the absorbance of Amide I + H_2_0 in the 1,646 cm^−1^ band and in Amide III corresponding to the 1,540 cm^−1^ band. The reduction in the absorbance of these chemical components is attributed to the change in the crystallinity, crystal size, and/or solubility of the crystals (Figure 3).

### 3.2. Maternal exposure to MeHg altered the crystalline structure in the mature enamel of rat incisors

**Figure 4:**
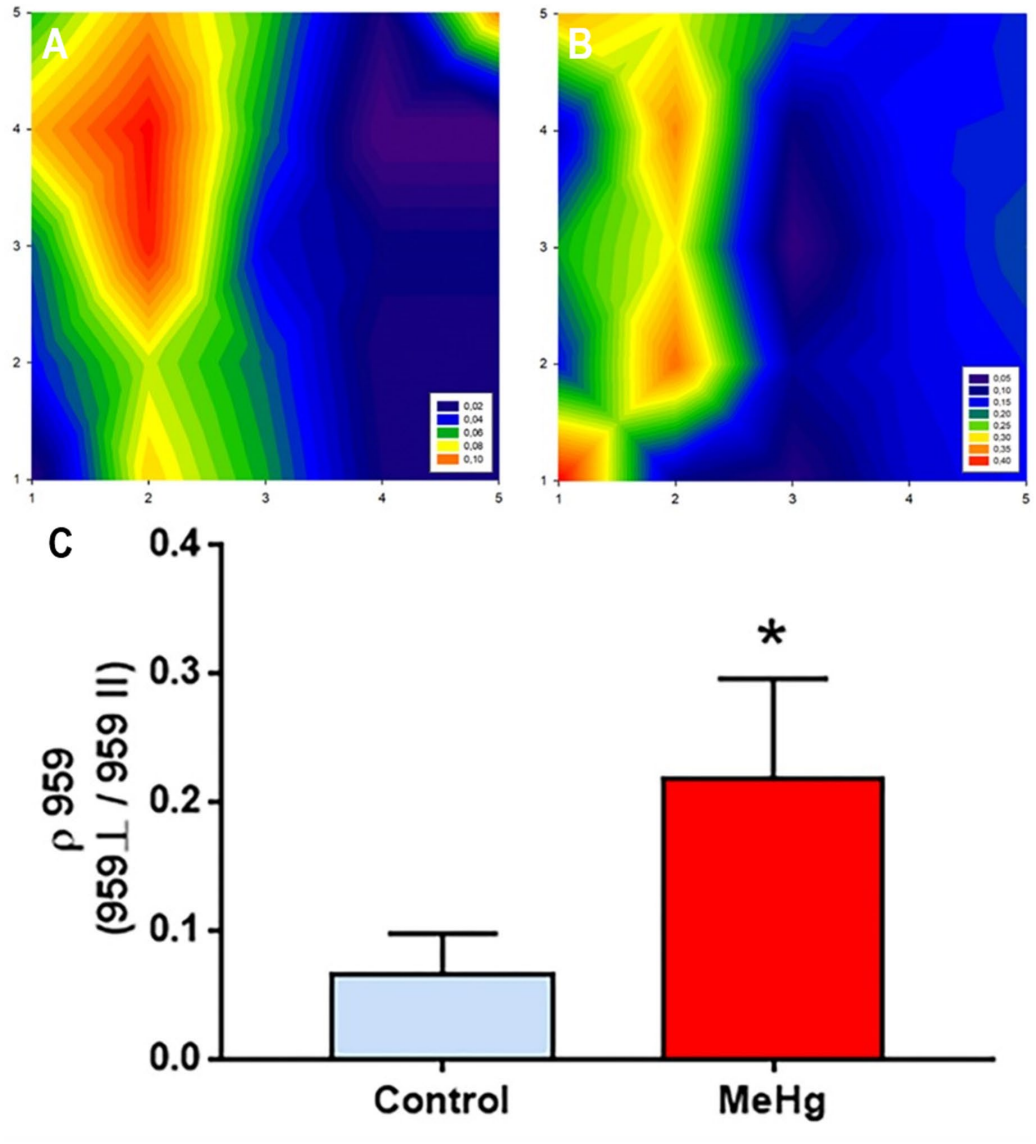
Effects of maternal MeHg exposure (40 μg/kg/day) and transfer to the offspring during the pre- and postnatal periods on the distribution of phosphate mineral content on the enamel surface generated by Raman analysis in the enamel of the incisors of offspring rats (41 days old, *n* = 10 per group). **(A)** A plot of the depolarization ratio obtained from the control group; **(B)** A plot of the depolarization ratio obtained from the exposed group. **(C)** A graph of the values related to the ratio between the perpendicular and parallel incidence of the phosphate band, the predominant chemical content in enamel. *Student’s *t*-test, *p* < 0.05.

The Raman spectra of enamel (Figure 4) were dominated by the symmetric stretching band of phosphate at ~959 cm^−1^. The distribution of depolarization ratio (ρ959) values in different areas of enamel were observed on the Raman mapping. The yellow/orange intensity observed in the control group (left side) is related to a phosphate highly polarization. On the other hand, in the exposed group (right side), a decrease in polarization was observed, as shown by the increase in blue colors. Furthermore, in the graphic below, the control enamel exhibited lower depolarization ratio values of ρ959 (0.065 ± 0.014) in comparison with those in the exposed enamel (0.218 ± 0.034; *p* = 0.0035; Figure 4).

**Figure 5:**
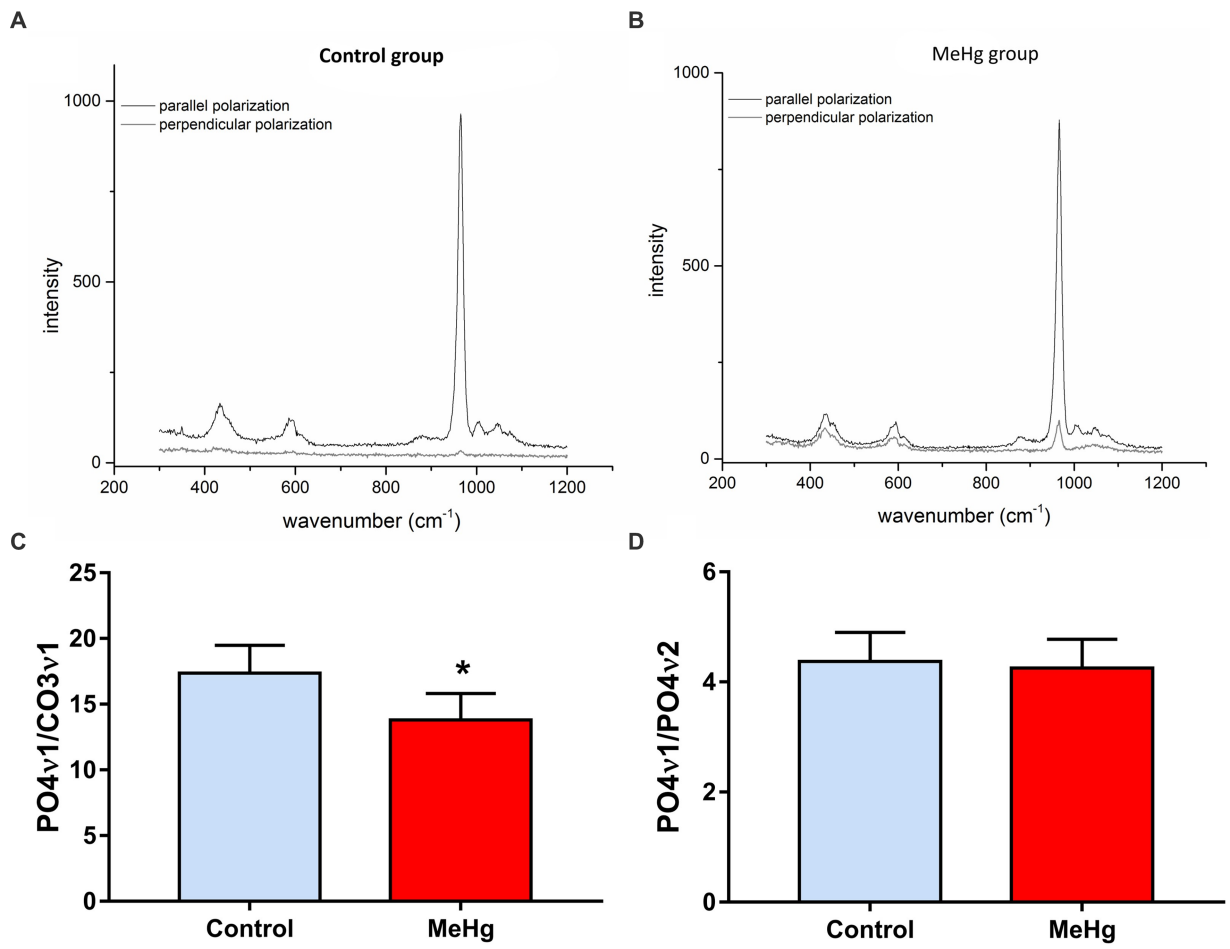
Effects of maternal MeHg exposure (40 μg/kg/day) and transfer to the offspring during the pre- and postnatal periods on the ratio of phosphate/phosphate and phosphate/carbonate mineral content on the enamel surface generated by Raman analysis in the enamel of the incisors of offspring rats (41 days old, *n* = 10 per group). **(A,B)** Comparison spectra of parallel and perpendicular polarized spectra for control and exposed groups. **(C,D)** a graph of the values related to the PO4ν1/CO3ν1 ratio and PO4ν1/PO4ν2 ratio. *Student’s *t*-test, *p* < 0.05.

Mineral content comparisons, regarding PO4ν1/CO3ν1 and PO4ν_1_/PO4ν_2_ ratio and revealed a decrease in the phosphate to carbonate ratio from (17.37 ± 5.17) in control group (13.80 ± 4.95, *p* = 0.02) to in exposed group, while the ν_1_ and ν_2_ phosphate modes did not exhibit significant changes between groups (control group: 4.37 ± 1.31; exposed group: 4.25 ± 1.29; *p* = 0.89; Figure 5).

### 3.3. Maternal exposure to MeHg causes ultrastructural changes in the mature enamel

**Figure 6:**
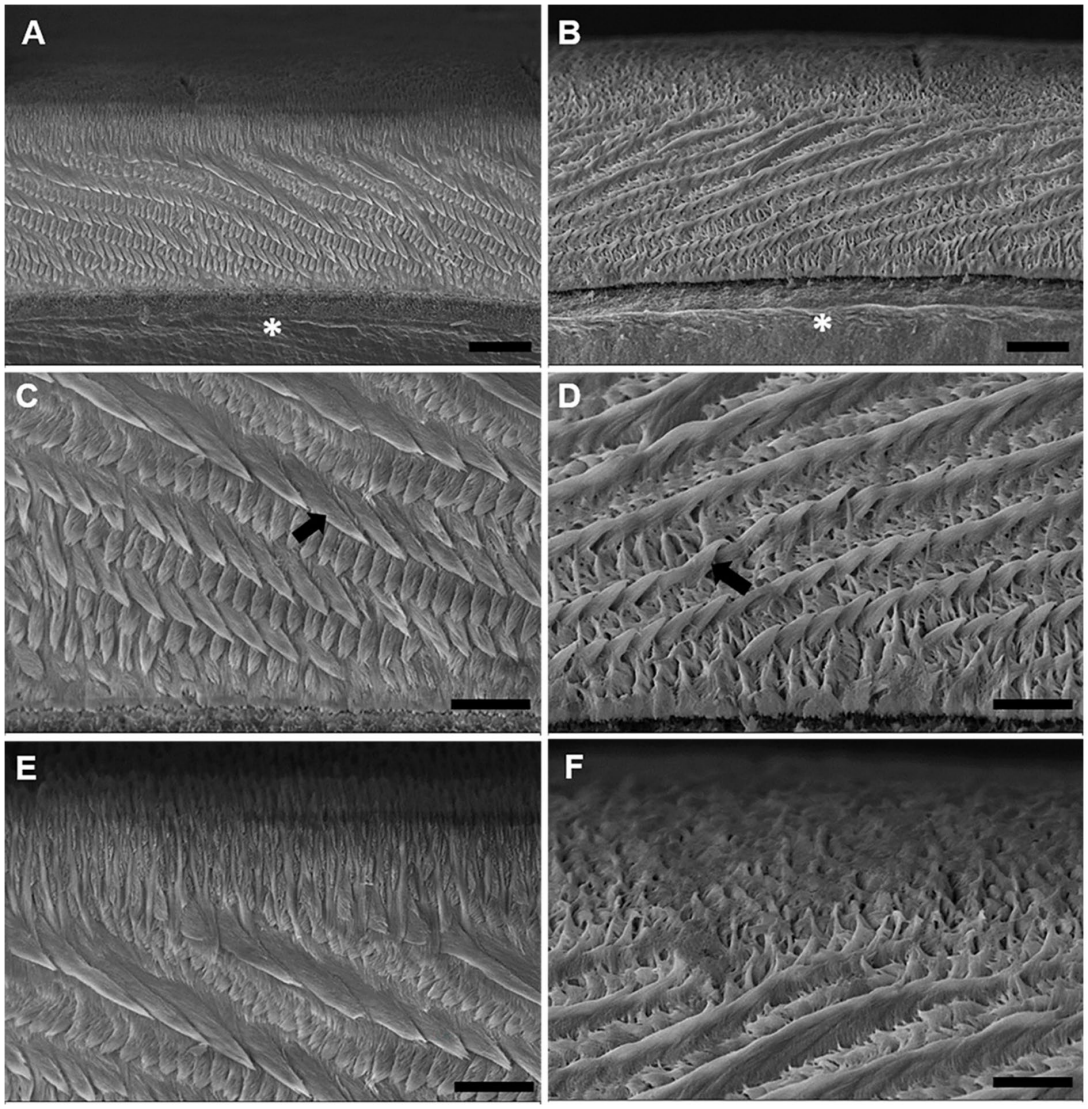
Effects of maternal MeHg exposure (40 μg/kg/day) and transfer to the offspring during the pre- and postnatal periods on morphological and structural aspects of the enamel of the incisors of offspring rats (41 days old). Electromicrographs of the cross-sectioned rat incisor. **(A,C,E)** Micrographs of the control group. **(B,D,F)** Electromicrographs of the group exposed to MeHg. **(A,B)** Is the enamel prismatic structure at 1500× magnification, where the entire thickness of the enamel is sectioned transversely. (*) indicate dentin. **(C,D)** at 4000× magnification, where the arrows indicate the enamel prisms. **(E,F)** the enamel surface region is found. Scale bar: **(A,B)** 10 μm; **(C–F)** 20 μm.

Analysis of enamel ultrastructure by scanning electron microscopy (Figure 6), revealed changes in the organization and integrity of the enamel prisms. As shown in electromicrographs B, D, and F, depicting the enamel of the exposed group, there was a change in the deposition and integrity of the enamel prisms compared with the electromicrographs of the exposed group (A, C, and E). In F, in the most superficial region of the enamel, a prismatic disorganization was noted (Figure 6).

### 3.4. Maternal MeHg exposure may induce physicochemical and structural alterations of mature dental enamel of the offspring by disturbing molecular order of secretory enamel organic extracellular matrix

**Figure 7:**
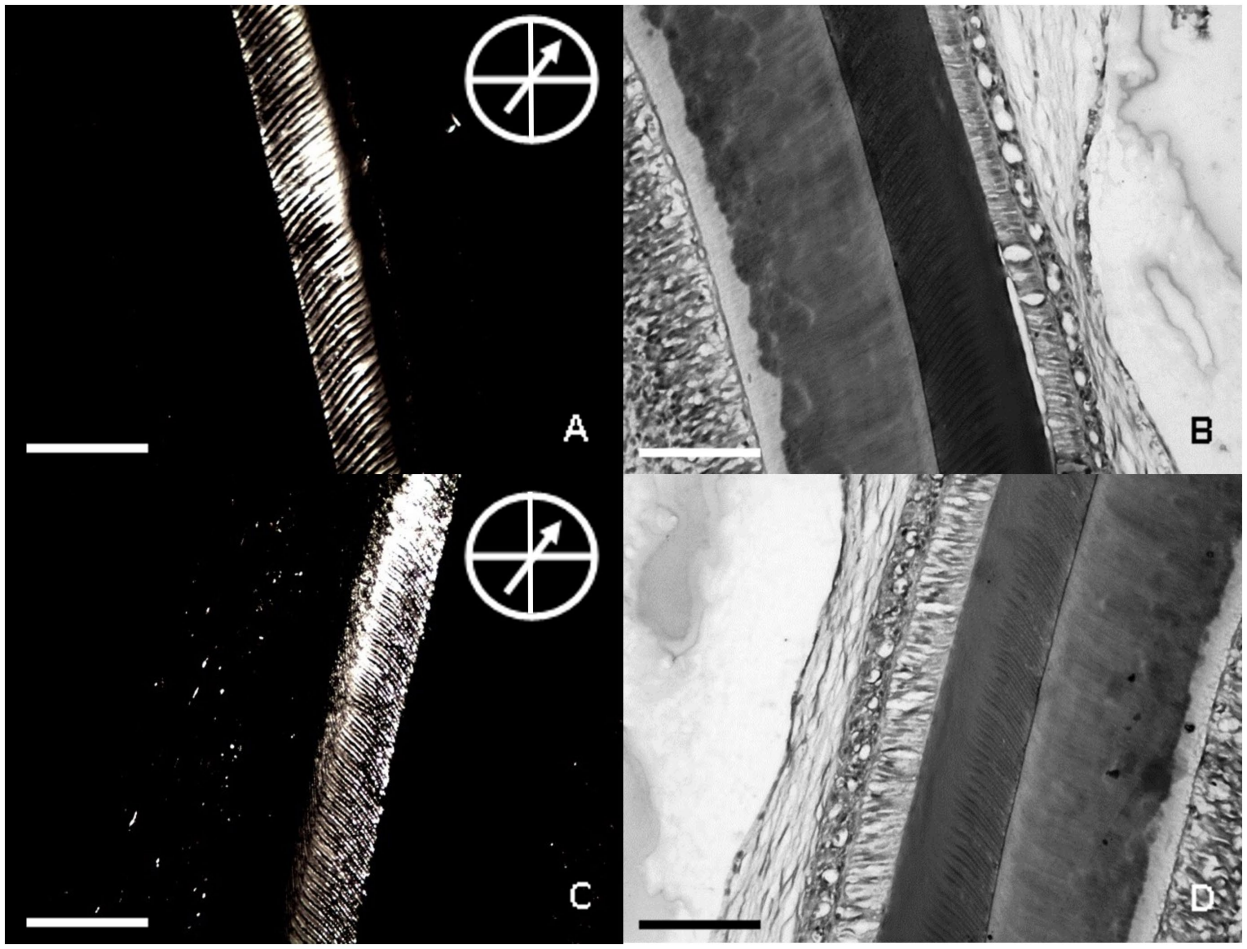
Effects of maternal MeHg exposure (40 μg/kg/day) and transfer to the offspring during the pre- and postnatal periods on morphological and structural aspects of the enamel of the incisors of offspring rats (41 days old, *n* = 10 per group). Bright-field light and polarizing light microscopies of the secretory stage EOECM of rats from control **(A,B)** and maternal MeHg transfer **(C,D)** groups. In the polarizing light micrograph, the analyzer is at 90° with the polarizer and the specimen exhibits position of maximum birefringence, as indicated by the arrow at 45° in relation to crossed bars. Bar of each micrograph represents 100 μm. **(A)** Birefringence of the secretory stage EOECM of enamel from control group. **(B)** Bright field of section **A**, after staining with HE. **(C)** birefringence of an unstained 5 μm thick section of the secretory stage EOECM of a rat upper incisor from MeHg group. **(D)** bright field of section C, after staining with HE.

MeHg did not induce evident morphological changes in the secretory stage EOECM, as revealed by BFLM; nevertheless, it is noteworthy that some animals from the exposed group exhibited a granular pattern of this matrix at TPLM, which is characteristically observed at the maturation stage of amelogenesis (Figure 7).

**Figure 8:**
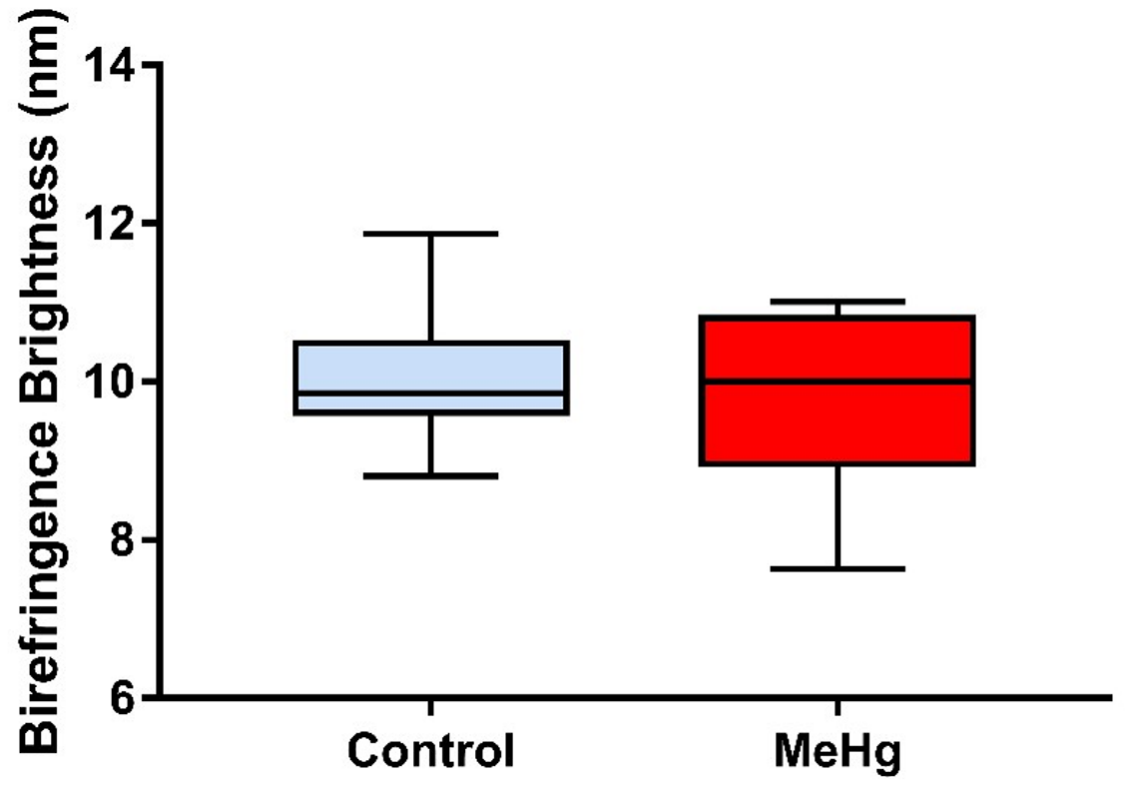
Effects of maternal MeHg exposure (40 μg/kg/day) and transfer to the offspring during the pre- and postnatal periods on morphological and structural aspects of the enamel of the incisors of offspring rats (41 days old, *n* = 10 per group). Optical retardations of birefringence brightness (nm) of unstained 5 μm thick sections of the secretory stage EOECM from control and MeHg groups (*p* > 0.05, Mann–Whitney test).

Maternal exposure to MeHg caused a slight decrease in optical retardation values of birefringence brightness of the secretory stage EOECM in the MeHg-exposed offspring group (median = 9.50; q1 = 8.63; q3 = 10.25; IQR = 1.62; *q* = quartile; IQR = interquartile range) compared to the control group (median = 9.86; q1 = 9.67; q3 = 10.18; IQR = 0.51), without statistical difference between the groups (*p* = 0.5995; Figure 8).

### 3.5. Maternal exposure to MeHg alters the resistance and hardness of the dental enamel of offspring

**Figure 9:**
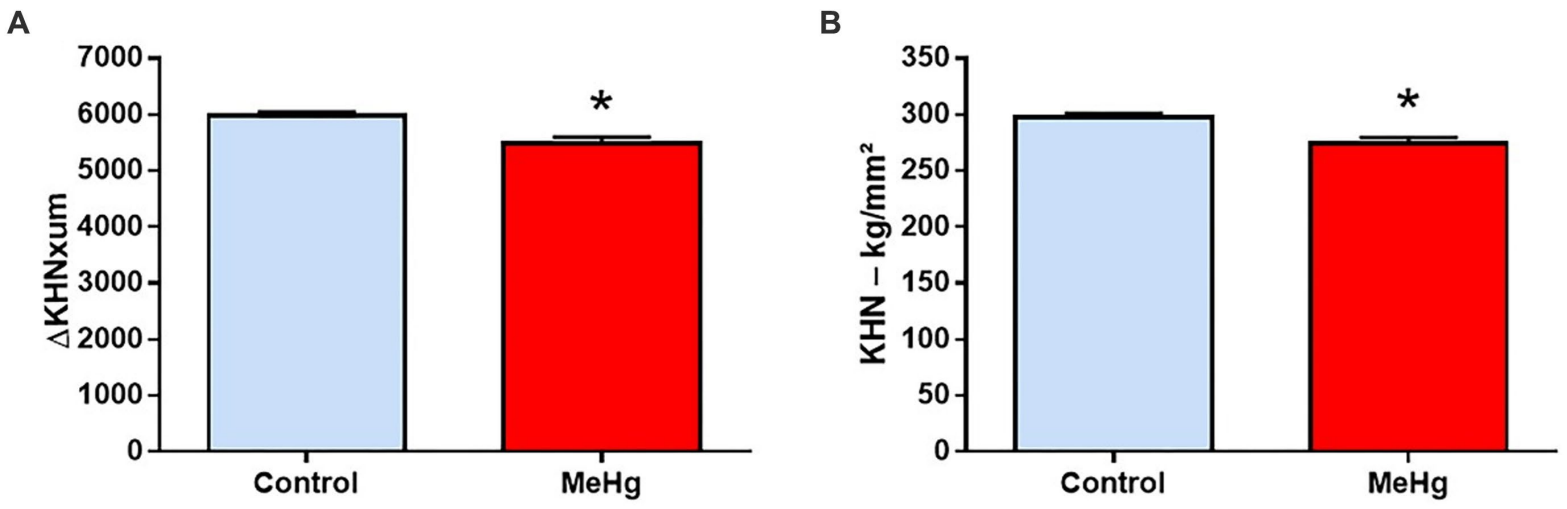
Effects of maternal MeHg exposure (40 μg/kg/day) and transfer to the offspring during the pre- and postnatal periods on hardness in the enamel of the incisors of offspring rats (41 days old, *n* = 10 per group). A representative panel of **(A)** integrated area of subsurface hardness (ΔKHNxum) and **(B)** Knoop hardness (KHN). Results are expressed as mean ± standard error. *Student’s *t*-test, *p* < 0.05.

Long-term exposure to MeHg caused a significant decrease in the mean (±SD) enamel integrated area of hardness in depth of the exposed group (5,478 ± 112) compared to the control group (5,982 ± 60.01; *p* = 0.001; Figure 9A). The same trend was observed for mean cross-sectional hardness, where there was a significant difference between the exposed (273.8 ± 5.40) and control groups (297.7 ± 3.12; p = 0.001; Figure 9B).

## 4. Discussion

In the present study, we evaluated the effects of gestational and lactational MeHg exposure on the physico-chemical, morphological, and microhardness properties in offsprings’ enamel. Our novel findings showed that MeHg exposure via maternal exposure was associated with changes in offspring enamel mineralization and crystallinity reflected by a reduction in the absorbance of phosphate and carbonate bands and increasing depolarization ratio of phosphate. Moreover, morphological analysis of the offspring enamel ultrastructure revealed that the organization and integrity of the prisms were affected by maternal MeHg exposure, concomitant with disturbances in molecular ordering of secretory enamel organic extracellular matrix as evidenced by transmitted polarizing light microscopy. Lastly, the maternal exposure was associated with significant alterations in the resistance and microhardness of offsprings’ enamel.

The exposure model employed a dose of 40 μg/kg/day, implemented by intake of cookies adulterated with MeHg (38, 39). Some aspects of this model deserve to be highlighted, given their valuable translational relevance. First, the chronic exposure of mothers via the oral pathway represents a physiological real-life model of exposure that mirrors exposures of human populations. As it has been already discussed (46), many *in vitro* and preclinical studies fail to recapitulate human exposures because of the different administration routes (disregarding the toxicokinetics characteristics of the exposure) and excessive dosing. In fact, considering the recently proposed allometric approach for mercury dosing of translational relevance (6), many studies have used excessively high doses of MeHg, triggering toxic mechanisms that may be of no translational relevance to exposed human populations. Second, we used a dose approximately equivalent to the Benchmark dose or LOAEL (Low Observed Adverse Effects Levels) estimated by the World Health Organization (with humans chronically exposed via contaminated-fish consumption presenting about 50 μg/g of hair mercury) (3) as described by Crespo-Lopez et al. (6), the weekly MeHg intake equivalent in rats was calculated as 215.83 μg/Kg, close to the dose used in our work (280 μg/Kg per week). Accordingly, our model simulates exposure of pregnant women, which triggers the first signals of neurological consequences of mercury. Furthermore, levels as high as 75 μg/g of hair mercury in adults have been recently described in Amazonian populations living in regions without ASGM influence (47, 48), supporting that these levels are readily found in human populations. Therefore, our findings are of translational relevance to vulnerable populations. Third, chronic MeHg consumption would cause a potential health risk, especially in children and pregnant women (49). Amazonian populations subside on fish, the main protein of the diet (50), and, therefore, their exposure to MeHg is a chronic problem. Recently, we have demonstrated that indirect exposure of the mother to MeHg causes damage to several brain regions, salivary glands and alveolar bone in the offspring (15, 21, 51).

MeHg is absorbed in the gastrointestinal tract and distributed to the blood. This metal forms soluble complexes mainly with thiol groups. While bile and feces are the major routes of excretion, breast milk is also a notable route in pregnant organisms (52). The offspring is commonly exposed during fetal life and during the breastfeeding period, followed by exposure in early childhood (53). MeHg can easily cross the placenta and absorbed by fetal tissues, leading to developmental alterations in children exposed *in utero* and early in life (54). High levels of proteins are found in breast milk, making it possible for these proteins to bind to metals. Therefore, breastfeeding can serve as a significant route of exposure to MeHg in infants (55).

Studies have shown that MeHg exposure during the prenatal period can alter the activity of key embryonic cellular signaling pathways (56, 57). In calcified long tissues, a study demonstrated that prenatal exposure to MeHg led to a significant delay in the development of different components of the appendicular skeleton of rat fetuses, such as delays in the formation of ossification centers and decreased growth of long bones, altering bone mineral density and content (58). As in oral calcified tissues, maternal exposure to MeHg in alveolar bone was able to induce changes in mineral composition, cause histological damage to osteocytes and collagen associated with a decrease in the quantity and thickness of alveolar bone (21). Tooth enamel has also been shown to be a target of this exposure during its formation, as the highly regulated process called amelogenesis occurs during the developmental stages (59). It is during these phases that enamel matrix proteins are secreted, until their maturation (60). This secretion of enamel matrix proteins during the expansion and development of mineral crystals (59), are associated with the mechanical and structural properties of their surface (61). Disturbances of these enamel properties are more prevalent in the deciduous dentition, during childhood, due to biological imbalances affecting cells involved in enamel formation and maturation (62, 63), as well as enamel quality could possibly play a role as a possible indirect marker of the harmful consequences of exposure to environmental agents.

Given the difficulty in obtaining human tooth samples during the early stages of enamel formation, we focused on rat incisors as they are especially useful given their continuous growth permitting analysis throughout all stages of enamel formation (25, 59, 64).

MeHg exposure in mothers modifed the mineral components in offspring enamel, phosphate and carbonate compounds, as well as the crystalline structure. Enamel is a highly mineralized tissue, containing 92–96% inorganic components, 1–2% organic matter, and 3–4% water. The inorganic component is mainly represented by calcium phosphate crystals in the form of hydroxyapatite (HAp) (23). The organic material contains proteins, mainly amelogenin, ameloblastin, and tuftelin, which play a role in the elasticity, viscoelasticity, and hardness of mineralized tissues, and by scarce amounts of lipids and carbohydrates [Qamar et al., 2021; (65)]. Reduced absorbance of phosphate, carbonate and amide components in dental enamel structure, observed in FTIR-ATR, may indicate a change in enamel composition and ultrastructure, which may affect its physical and mechanical properties (66). Thus, the reduction in absorbance of these compounds may be an indication of the decrease in the amount of them present in enamel, and the variation in mineral composition of dental enamel. Further evaluation of the phosphate to carbonate ion revealed a decrease of this ratio, meaning a higher decrease of phosphate ion. The presence of carbonate ions in enamel has been shown to have a significant effect on the structure and properties of enamel. Carbonate ions can substitute for phosphate ions in the hydroxyapatite lattice, leading to changes in crystal structure, solubility, and mechanical properties (67) determined a decrease in enamel crystallinity concomitant with an increase of carbonate content. A reduced phosphate to carbonate ratio in enamel, with the formation of an irregular surface, can be an indication of a less resistant and more friable weaker and vulnerable enamel structure.

In addition to its mineral structure, we found morphological changes in the enamel. The dominant characteristic of enamel on a microscopic scale is enamel prisms (65). In the histological structure of the enamel observed in this study, maternal exposure to MeHg caused a change in the organization and integrity of the prisms of the offspring’s incisors. The rat incisor has a lamellar pattern of uniseriate prisms in the internal enamel and incisally parallel prisms directed to the external enamel (24, 64). Each prism consists of a set of crystalline apatite structures that are aligned parallel to each other and maintained as a cohesive unit (65). In this way, prismatic disorganization has the potential of compromising molecular functions (25) of developmental enamel, such as phosphate and ion transport, movement of ameloblasts, remodeling of junctional complexes of these cells during amelogenesis, factors that influence enamel prismatic architecture (25).

Transmitted polarizing light microscopy reveals tissue molecular order with possible functional relevance in common unstained histological sections. This is the first report on the effects of MeHg via maternal transfer, on the birefringence of secretory stage EOECM which showed maximum brightness when enamel rods were oriented at 45° with respect to the polarizer and analyzer filters in exposed and control samples. Most MeHg rats did not present expressive changes in morphological and anisotropic properties of their secretory enamel matrices, as revealed by TPLM and BFLM. A granular pattern of some of those matrices was noted by TPLM (Figure 7C), which is characteristically observed at the maturation stage of amelogenesis and means extensive proteolysis (68). These results indicate that mature enamel alterations induced by maternal MeHg transfer may be preceded by important alterations in aggregational features of the secretory stage EOECM and in the orientation of its prismatic components. The enamel is characterized by anisotropic mechanical properties, which collectively ensure the efficiency of the mastication process and functional stress, which are influenced by the orientation and structural organization of the enamel organic matrix during amelogenesis (69).

Along these lines, maternal exposure to MeHg also leads to changes in the mechanical properties of the mineralization functional patterns in offspring. An association was observed with the metal administered and a decrease in the resistance and microhardness of the enamel surface. This may be associated with changes in the mineral, structural, and physicochemical properties of the enamel, as previously observed. Indentation studies on dental enamel have provided insights into dental enamel materials properties and behavior under mechanical loading (70). Defects in the quality and quantity of tooth enamel caused by disturbance during the developmental phase, as well as the extent and duration of the insult, are usually expressed by tooth opacity, hypoplasia, hypomineralization, and hypomaturation (71).

Our data demonstrate that mechanical properties reside in complex levels of structural organization that, in turn, are the result of a highly coordinated and mediated matrix mineralization process that requires organic and inorganic components. Our results revealed changes in the physicochemical properties of offspring’s enamel, and that these changes may be preceded by molecular disturbances of the secretory stage EOECM. Thus, alterations were observed in hardness and ultrastructure, leading us to conclude that, during amelogenesis, the enamel of the offspring is more susceptible to the effects of maternal exposure to MeHg during pregnancy and breastfeeding. Moreover, from a translational perspective, the findings show that early exposure to MeHg can be a modulator of dental integrity in populations vulnerable to MeHg, pointing to the need for investigation in human populations of the possible association between early mercury exposure and higher prevalence of dental damage.

## Data availability statement

The original contributions presented in the study are included in the article/supplementary material, further inquiries can be directed to the corresponding author.

## Ethics statement

The animal study was reviewed and approved by Ethics Committee on Animal Use No. 8613011217 (CEUA/UFPA last approval date in 11/22/2019).

## Author contributions

VC, PN, and LB: conceptualization, data curation, investigation, methodology, and writing–original draft. MP and AA: formal analysis, data curation, and software. AD, JP, RA, AE, and SP: formal analysis, investigation, data curation, and writing–review and editing. MC-L and MA: formal analysis, investigation, and writing–review and editing. RL: conceptualization, formal analysis, investigation, methodology, supervision, and writing–review and editing. All authors contributed to the article and approved the submitted version.

## Funding

RL is a researcher from Conselho Nacional de Desenvolvimento Científico e Tecnológico (CNPq) and received grant under number 312275/2021–8. MC-L is also recognized by the CNPq as highly productive researcher with the grant number 313406/2021–9. Also, this research was funded by PROCAD Amazônia–CAPES (23038.005350/2018–78). The APC was funded by Pró-Reitoria de Pesquisa e Pós-graduação from Federal University of Pará (PROPESP-UFPA). The funders had no role in study design, data collection and analysis, decision to publish, or preparation of the manuscript.

## Conflict of interest

The authors declare that the research was conducted in the absence of any commercial or financial relationships that could be construed as a potential conflict of interest.

## Publisher’s note

All claims expressed in this article are solely those of the authors and do not necessarily represent those of their affiliated organizations, or those of the publisher, the editors and the reviewers. Any product that may be evaluated in this article, or claim that may be made by its manufacturer, is not guaranteed or endorsed by the publisher.
